# Erosive Pustular Dermatosis of the Scalp Following Hair Transplantation: A Rare Complication

**DOI:** 10.7759/cureus.79328

**Published:** 2025-02-19

**Authors:** Guillermo A Guerrero-González, Mayra A Reyes-Soto, Daniela López Quintero, Juana I Garza Chapa

**Affiliations:** 1 Dermatologic Surgery, Pango Dermatología, Monterrey, MEX; 2 Dermatology, Hospital Universitario “Dr. José Eleuterio González”, Monterrey, MEX; 3 Dermatology, Hospital Universitario "Dr. José Eleuterio González", Monterrey, MEX

**Keywords:** erosive pustular dermatosis of the scalp, follicular unit extraction, hair transplantation, post-surgical inflammatory dermatoses, scalp dermatosis

## Abstract

Erosive pustular dermatosis of the scalp (EPDS) is a rare inflammatory condition characterized by sterile pustules, crusts, and skin atrophy, leading to scarring alopecia. While typically affecting the elderly, this report discusses a rare instance in a 31-year-old male who developed EPDS following follicular unit extraction (FUE) for androgenetic alopecia. The diagnosis was confirmed through clinical, dermoscopic, and histopathological examination. Initial treatment with topical steroids showed temporary improvement, but the condition relapsed upon tapering. High-potency topical corticosteroids were ultimately effective. This case underscores the importance of early recognition of EPDS, highlighting the value of clinical, trichoscopic, and histopathologic examination in improving patient outcomes. It also expands the understanding of EPDS by suggesting that surgical trauma may trigger pathergy, potentially initiating the condition in younger individuals without typical risk factors. Given the increasing popularity of hair transplantation, clinicians must remain vigilant about rare complications like EPDS to ensure prompt diagnosis and prevent irreversible alopecia.

## Introduction

Erosive pustular dermatosis of the scalp (EPDS) was first described by Burton and Pye in 1979, who documented persistent pustular lesions leading to scarring alopecia in six elderly women [1]. EPDS predominantly affects the elderly due to age-related skin changes, such as atrophy that weakens the protective barrier and cumulative sun damage. Additionally, preexisting scalp conditions common in this population, such as actinic keratosis and androgenetic alopecia (AGA), contribute to a vulnerable scalp environment. Older adults are also more likely to undergo scalp surgeries to remove skin cancers, which result from chronic sun exposure. This surgical trauma further compromises the scalp, triggering EPDS [2]. It is a rare inflammatory condition affecting the scalp, characterized by sterile pustules, crusts, and skin atrophy, and typically follows a chronic, undulating course marked by partial improvement without complete resolution. In the absence of appropriate treatment, the condition may progress to severe scarring, leading to cicatricial alopecia [3].

Iatrogenic factors, such as cryotherapy, carbon dioxide lasers, topical chemotherapy, as well as malignancies, infections, drugs, and surgical interventions, including grafting, are known triggers [2-4]. The pathogenesis has not been fully established, but trauma is thought to develop an abnormal immune response against hair follicle structures, resulting in an inflammatory cascade and subsequent fibrosis of the follicle [3]. EPDS has been observed in patients with autoimmune conditions like rheumatoid arthritis, autoimmune hepatitis, Hashimoto's thyroiditis, collagen vascular disease, Takayasu's arteritis, and myasthenia gravis [5]. High-potency topical steroids have been proven effective as a first-line treatment [6].

Differential diagnoses include squamous cell carcinoma and basal cell carcinoma; bullous autoimmune diseases such as cicatricial pemphigoid; subcorneal pustular dermatosis and pustular psoriasis; eczema; herpes zoster; pyoderma, folliculitis and kerion; as well as other scarring alopecias, including dissecting cellulitis and folliculitis decalvans [3].

While EPDS is most commonly seen in the elderly with pre-existing scalp conditions, there is an increasing recognition of its occurrence in younger individuals, especially those undergoing scalp-related surgical interventions like follicular unit extraction (FUE), a popular procedure for AGA. To our knowledge, only a few cases of EPDS following hair transplantation have been reported. Here, we present a 31-year-old male who developed EPDS after hair transplantation, despite lacking the typical predisposing factors, highlighting the importance of early diagnosis and tailored treatment to prevent irreversible complications.

## Case presentation

**Figure 1:**
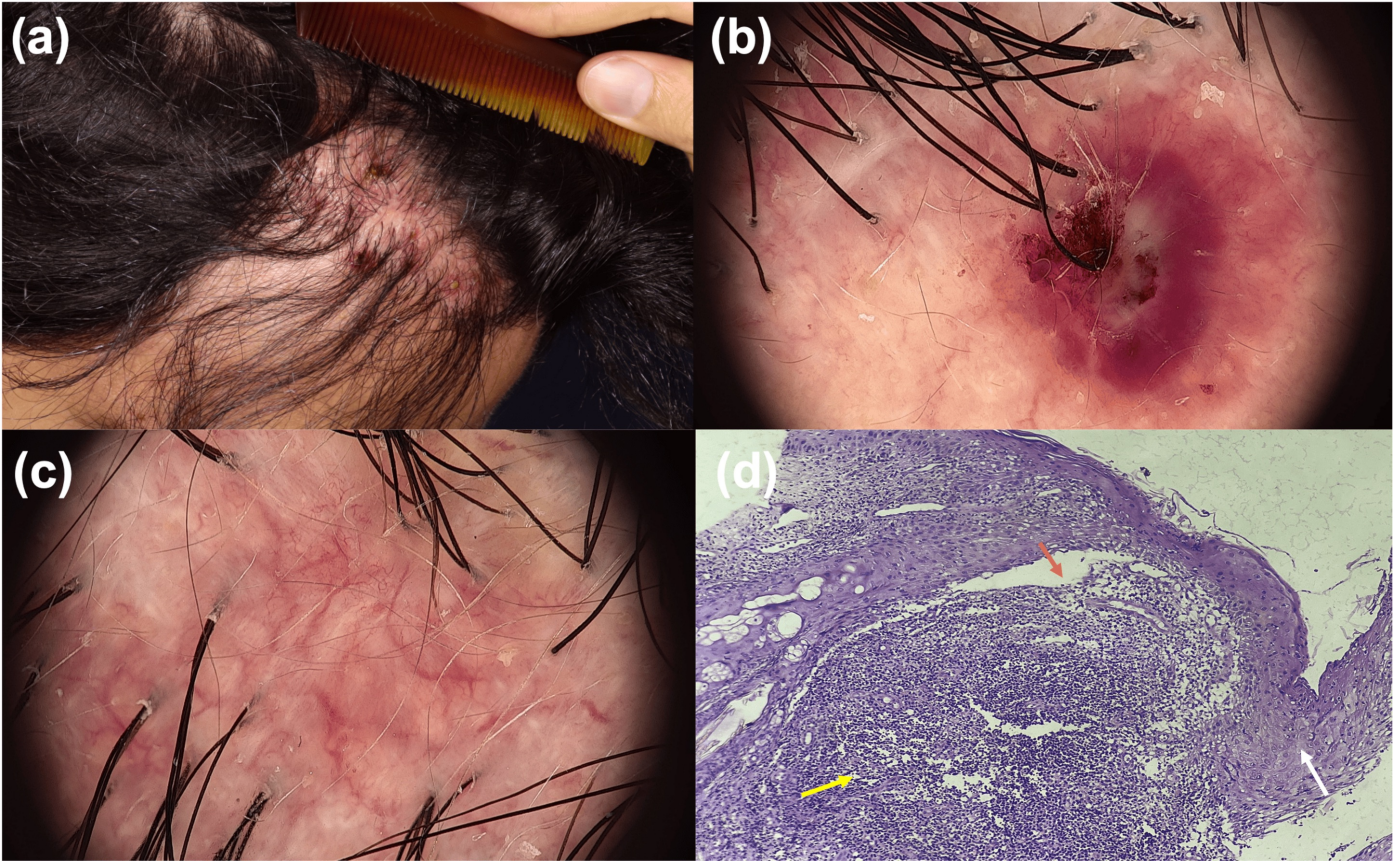
Clinical and trichoscopic manifestations of EPDS following hair transplantation. (a) Erythematous plaque with crusting and pustules in the recipient area. (b) Trichoscopic images revealing a pustule with crust and erythema. (c) Atrophy with visible dermal vessels and scarring. (d) Histopathologic manifestations of EPDS following hair transplantation. Histopathology stained with hematoxylin and eosin (H&E) 10x, showing acanthosis (white arrow), a subcorneal pustule (orange arrow), accompanied by mixed inflammation of lymphocytes and neutrophils in the dermis (yellow arrow). EPDS: Erosive pustular dermatosis of the scalp

A 31-year-old male, presented with an 18-month history of an asymptomatic, crusted erythematous plaque on the frontal area, which developed one year after undergoing FUE for AGA. He had no significant medical conditions or history of cosmetic use, and no notable side effects were observed during the follow-up period. Scalp examination revealed erythematous plaque, ulcerations with yellow crusting, and pustules in the recipient area (Figure 1a). Trichoscopy showed erythematous areas, pustules, crusting, atrophy, and visible dermal vessels (Figures 1b-1c). The rest of the scalp appeared normal. The culture showed no bacterial or fungal growth. Complete blood count, blood chemistry, erythrocyte sedimentation rate, and C-reactive protein were within normal limits. A scalp biopsy from the frontal area showed acanthosis, subcorneal pustules, and a mixed inflammatory infiltrate with lymphocytes, macrophages, and neutrophils. The correlation of the clinical history, histopathology, and cultures established the diagnosis of EPDS (Figure 1d).

The patient had initially been treated with topical corticosteroids, which led to partial improvement. However, the lesion relapsed following the tapering of treatment. Given the recurrence, a more comprehensive treatment approach was pursued. The patient was subsequently prescribed topical 0.1% tacrolimus and oral isotretinoin (20 mg/day) for six months. Despite this regimen, there was no significant clinical improvement. High-potency topical corticosteroids were initiated for one month, resulting in a substantial improvement and eventual resolution. Tapering of topical steroids was made with no relapses. After one year, adequate graft growth was achieved with no recurrences of EPDS.

## Discussion

Hair transplantation is a widely practiced procedure, especially in patients with AGA. While generally successful with favorable regrowth outcomes, inflammatory complications, though rare, can occur. These complications, including conditions such as lichen planopilaris (LPP) and EPDS, have been reported in only 0.08% of cases [7].

EPDS following hair transplantation presents a unique clinical challenge, highlighting the importance of early recognition and intervention to prevent scarring alopecia. Although rare, it may be underreported, particularly in younger patients, who are not the typical demographic for EPDS. This case underscores that EPDS can occur in younger individuals with minimal sun damage, suggesting that the inflammatory response triggered by hair transplantation might be sufficient to initiate EPDS. Previous studies have reported EPDS following surgical procedures. Saridakis et al. documented 13 cases of post-surgical EPDS, noting that 73.7% appeared at or near the surgical site, with only one case following hair transplantation [4,8].

To our knowledge, only four cases of EPDS as a complication of hair transplantation have been reported (Table 1) [4,7,9]. The average onset time after hair transplantation was 10.1 months. In one case, grafts were placed in the vertex area, with the occipital area serving as the donor site in all cases. None of the patients had a history of scalp trauma, autoimmune diseases, or relevant personal or family medical history. All patients experienced resolution without relapses. Only one reported inadequate graft growth (<50%) and subsequently underwent a second hair transplant [7].

**Table 1:** Clinical information of the cases of erosive pustular dermatosis of the scalp following hair transplantation. ND: no data; BID: twice a day

Author (year)	Gender/age	Timing of onset	Clinical presentation/site	Symptoms	Trichoscopy	Histopathological findings	Treatment	Outcome
Our case	Male, 31 years	12 months	Erythematous plaque with pustules and crusts; frontal scalp	Asymptomatic	Pustules, crust, scarring, and dermal vessels	Epidermis with erosions, acanthosis, and subcorneal pustules, dense dermal infiltrate with numerous lymphocytes, macrophages, and neutrophils	High-potency topical steroid	Resolution
Shahmoradi et al. (2014) [4]	Male, 35 years	9 months	Diffuse crusting associated with multiple pustular, exudative, and erosive lesions; parietal scalp	Mild burning sensation	ND	Dense dermal infiltrate of neutrophils and lymphocytes, mainly perifollicular	Topical clobetasol propionate lotion, BID	Resolution
Saad et al. (2022) [7]	Male, 32 years	12.4 months (average)	Pustulo-crusty lesions in the recipient area; frontal, temporal, and parietal scalp	Itching	Crusts, pustules, erythema, and rarefactions of the follicular ostia	Not confirmative	ND	Resolution
	Male, 61 years	12.4 months (average)	Pustulo-crusty lesions in the recipient area; frontal, temporal, and parietal scalp	Itching	Rarefaction of follicular ostia, perifollicular erythema, pustules, and crusts	Suppurative inflammatory rearrangement of hair follicles associated with marked fibrosis and sometimes cystic dystrophy	ND	Resolution
El Kabbaj et al. (2005) [9]	Male, 75 years	3 to 7 months	Oozing erosions associated with crusts and pustular elements; vertex	ND	ND	Epidermal and dermal inflammatory infiltrate made up of polynuclear neutrophils sometimes associated with plasma cells but without individualized pustule	Silver sulfadiazine and cerium nitrate for two months followed by oral zinc gluconate 30 mg/day and a class 2 topical steroid	Resolution

The pathogenesis of EPDS after hair transplantation remains unclear. Some authors consider EPDS part of the spectrum of inflammatory dermatoses [10], while others propose an autoimmune etiology, where physical trauma to the skin induces the production of autoantibodies targeting epidermal and dermal structures, leading to a secondary inflammatory reaction [11]. EPDS has been observed in patients with autoimmune conditions like rheumatoid arthritis, autoimmune hepatitis, Hashimoto's thyroiditis, collagen vascular disease, Takayasu's arteritis, and myasthenia gravis [5]; however, the underlying autoimmune mechanisms and autoantibodies remain uncertain and need further investigation [12]. Trauma associated with FUE may disrupt the epidermal barrier, triggering an aberrant immune response [2,4,13]. This response involves chronic immunologic dysregulation, characterized by abnormal neutrophil chemotaxis and cytokine production against epidermal or follicular antigens. Elevated levels of interleukin-8 (IL-8), known for its potent attraction of neutrophils and lymphocytes, have been observed in EPDS patients with rheumatoid arthritis and myasthenia gravis [14,15]. EPDS shares clinicopathologic features with pyoderma gangrenosum (PG), a neutrophilic dermatosis where minor trauma triggers an exaggerated inflammatory response, leading to pustules and erosions at surgical sites. The clinical and histopathological similarities suggest shared pathogenic pathways, with pathergy likely playing a central role in both conditions [5,16].

The delayed onset of clinical symptoms - emerging over a year after the initial trauma - suggests a prolonged, dysregulated inflammatory response, which can continue long after the initial trigger, ultimately leading to granulation tissue formation and scarring alopecia. The favorable response to corticosteroids further supports an inflammation-driven process [14].

Histopathological examination is characterized by epidermal atrophy and ulceration; the dermis shows a dense inflammatory infiltrate composed of a variable amount of neutrophils, lymphocytes, and occasional plasma cells, with variable degrees of fibrosis and granulation tissue formation [1]. However, these findings are non-specific. Cultures are typically negative; however, positive results likely reflect secondary colonization rather than primary infection, given the poor response to antibiotic therapy. Blood tests are usually unremarkable, mild leukocytosis or elevated inflammatory markers may occasionally be observed [11]. Due to its lack of specificity, the diagnosis of EPDS is based on clinicopathological correlation and exclusion of other potential diagnoses. A comprehensive differential diagnosis should be considered, including common eczema, folliculitis, kerion, PG, squamous cell carcinoma, basal cell carcinoma, pemphigus, cicatricial pemphigoid, pustular psoriasis, subcorneal pustular dermatosis, and scarring alopecias, particularly dissecting cellulitis and folliculitis decalvans [11,17].

Treatment with high-potency topical corticosteroids is the first-line treatment, achieving an 83% resolution rate [6]. Topical calcineurin inhibitors, topical calcipotriol, and topical treatment with dapsone 5% gel were likewise shown to be effective [3]. The following treatment regimens have been reported to be effective: non-steroidal anti-inflammatory drugs (NSAIDs), retinoids, systemic antibiotics, oral zinc, and systemic corticosteroids [6]. Photodynamic therapy and doxycycline are reserved for resistant EPDS cases or when conventional treatments are contraindicated [5,18].

The prognosis of EPDS is generally favorable with early recognition and appropriate management, though delayed treatment may lead to scarring alopecia and chronic inflammation [6].

## Conclusions

A thorough clinical and trichoscopic examination prior to hair transplantation is essential to identify underlying inflammatory conditions that may predispose patients to complications. As hair transplants become increasingly common, long-term follow-up is crucial to detect and manage rare but significant complications such as EPDS. Given the challenging nature of EPDS management, a multidisciplinary approach involving dermatologists, trichologists, and hair transplant surgeons is necessary to optimize outcomes. Increased awareness among clinicians and the incorporation of standardized screening protocols before hair transplantation may help mitigate risks and improve patient safety. Future research should focus on identifying predisposing factors and developing evidence-based guidelines for prevention and early intervention in post-transplant inflammatory scalp disorders.

## References

[1] Pye RJ, Peachey RD, Burton JL (1979). Erosive pustular dermatosis of the scalp. Br J Dermatol.

[2] Starace M, Loi C, Bruni F, Alessandrini A, Misciali C, Patrizi A, Piraccini BM (2017). Erosive pustular dermatosis of the scalp: clinical, trichoscopic, and histopathologic features of 20 cases. J Am Acad Dermatol.

[3] Wilk M, Zelger BG, Hauser U, Höpfl R, Zelger B (2018). Erosive pustular dermatosis of the scalp: reappraisal of an underrecognized entity. J Dtsch Dermatol Ges.

[4] Shahmoradi Z, Abtahi-Naeini B, Pourazizi M (2014). Erosive pustular dermatosis of the scalp following hair transplantation. Adv Biomed Res.

[5] Bhargava S, Yumeen S, Henebeng E, Kroumpouzos G (2022). Erosive pustular dermatosis: delving into etiopathogenesis and management. Life (Basel).

[6] Junejo MH, Kentley J, Rajpopat M, Tan XL, Mohd Mustapa MF, Harwood CA (2021). Therapeutic options for erosive pustular dermatosis of the scalp: a systematic review. Br J Dermatol.

[7] Saad S, Cavelier-Balloy B, Smadja J, Assouly P, Reygagne P (2022). Inflammatory complications after hair transplantation: report of 10 cases. J Cosmet Dermatol.

[8] Saridakis S, Giesey RL, Ezaldein HH, Scott JF (2020). Erosive pustular dermatosis of the scalp following surgical procedures: a systematic review. Dermatol Online J.

[9] El Kabbaj N, Dereure O, Guillot B (2005). Erosive pustulosis of the scalp: 3 cases [Article in French]. Ann Dermatol Venereol.

[10] Michelerio A, Vassallo C, Fiandrino G, Tomasini CF (2021). Erosive pustular dermatosis of the scalp: a clinicopathologic study of fifty cases. Dermatopathology (Basel).

[11] Mastroianni A, Cota C, Ardigò M, Minutilli E, Berardesca E (2005). Erosive pustular dermatosis of the scalp: a case report and review of the literature. Dermatology.

[12] Watanabe S, Takizawa K, Hashimoto N, Ishibashi Y (1989). Pustular dermatosis of the scalp associated with autoimmune diseases. J Dermatol.

[13] Miteva M (2021). Hair Pathology with Trichoscopic Correlations.

[14] Yamamoto T, Furuse Y (1995). Erosive pustular dermatosis of the scalp in association with rheumatoid arthritis. Int J Dermatol.

[15] Sawada Y, Bito T, Kawakami C, Shimauchi T, Nakamura M, Tokura Y (2010). Erosive pustular dermatosis of the scalp and leg associated with myasthenia gravis: a possible pathogenetic role for neutrophil-stimulating cytokines and chemokines. Acta Derm Venereol.

[16] Tomasini C, Michelerio A (2019). Erosive pustular dermatosis of the scalp: A neutrophilic folliculitis within the spectrum of neutrophilic dermatoses: a clinicopathologic study of 30 cases. J Am Acad Dermatol.

[17] Piccolo V, Russo T, Bianco S, Ronchi A, Alfano R, Argenziano G (2019). Erosive pustular dermatosis of the scalp: why do we miss it?. Dermatology.

[18] Maglie R, Quintarelli L, Caproni M, Antiga E (2019). Impressive response of erosive pustular dermatosis of the scalp to lymecycline monotherapy. J Dtsch Dermatol Ges.

